# Pre-conception maternal erythrocyte saturated to unsaturated fatty acid ratio predicts pregnancy after natural cycle frozen embryo transfer

**DOI:** 10.1038/s41598-018-19500-0

**Published:** 2018-01-19

**Authors:** Christopher C. Onyiaodike, Heather M. Murray, Ruiqi Zhang, Barbara J. Meyer, Fiona Jordan, E. Ann Brown, Robert J. B. Nibbs, Helen Lyall, Naveed Sattar, Scott M. Nelson, Dilys J. Freeman

**Affiliations:** 1 School of Medicine, University of Glasgow, Glasgow, UK; 2 Robertson Centre for Biostatistics, University of Glasgow, Glasgow, UK; 3 School of Medicine, Metabolic Research Centre, University of Wollongong, Wollongong, NSW Australia; 4 Institute of Cardiovascular and Medical Sciences, University of Glasgow, Glasgow, UK; 5 Institute of Infection, Immunity and Inflammation, University of Glasgow, Glasgow, UK

## Abstract

The environment for embryo implantation and fetal growth and development is affected by maternal nutritional, metabolic and health status. The aim of this prospective, cohort study was to test whether plasma metabolic and inflammatory biomarkers can predict pregnancy resulting from *in vitro* fertilisation (IVF). Women with a natural menstrual cycle undergoing frozen embryo transfer (FET) were recruited and fasting baseline blood samples were collected a mean of 3.4 days prior to the luteinising hormone (LH) surge and a non-fasting blood sample was taken on the day of FET. Ongoing pregnancy was defined by positive fetal heartbeat on ultrasound scan at day 45 post LH surge. Thirty-six pregnancies resulted from FET in 143 women. In an overall stepwise multivariable analysis, erythrocyte saturated to unsaturated fatty acid ratio was positively associated with ongoing pregnancy. A similar model incorporating day of FET covariates found that erythrocyte saturated to unsaturated fatty acid ratio, erythrocyte fatty acid average chain length and plasma log-triglycerides predicted ongoing pregnancy. In conclusion, a higher peri-conceptional saturated to unsaturated fatty acid ratio predicted ongoing pregnancy after natural cycle frozen embryo transfer and may reflect a maternal nutritional status that facilitates pregnancy success in this assisted conception scenario.

## Introduction

The environment for embryo implantation and fetal growth and development is affected by maternal nutritional, metabolic and health status. Pre-pregnancy body mass index (BMI), to a greater extent than gestational weight gain or dietary intake, influences the maternal metabolic adaptation to pregnancy highlighting the importance of peri-conceptional health^1^. In women undergoing assisted reproduction, maternal metabolic health can influence the success of treatment. For example, maternal obesity can affect oocyte and embryo quality^2^, uterine receptivity^3^ and ultimate success of *in vitro* fertilisation (IVF)^4^.

Promotion of a healthy pregnancy in women prior to conception is a potential approach to reduce perinatal morbidity and mortality, however evidence for good biomarkers of peri-conceptional health is lacking. The aspiration is to find new predictive markers that, prior to pregnancy, can identify women at risk of an adverse outcome and identify those who could benefit from lifestyle intervention to improve their peri-conceptional health in both natural conception and IVF scenarios. It has been suggested that metabolites present in the plasma may reflect the environment in the ovary^5^. Interestingly, the type of serum fatty acid on day of oocyte retrieval has been associated with pregnancy success with high serum α-linolenic acid levels linked to a lower chance of pregnancy^6^. The blood fraction in which fatty acids reside inform us of different aspects of fatty acid metabolism. Plasma fatty acids, predominately derived from phospholipids in lipoproteins, represent plasma fatty acid flux or turnover and are heavily influenced by fasting status. Erythrocyte fatty acid content represents an integrative measure of whole body fatty acid status over the preceding three months (the lifespan of an erythrocyte is 120 days) and erythrocytes may act as a potential storage vehicle for some key polyunsaturated fatty acids such as arachidonic acid (20:4n-6) and docosahexaenoic acid (22:6n-3)^7^. Metabolomic profiling has identified maternal plasma fatty acid composition as a potential mediator of the adverse effect of pre-pregnancy BMI on pregnancy outcome^1^. Thus markers of nutritional status (such as plasma and erythrocyte fatty acid composition)^8^, metabolic and inflammatory markers associated with maternal insulin resistance and obesity^9,10^ and inflammatory markers associated with pregnancy loss^11^ could be potentially informative for predicting pregnancy success.

The aim of this study was to test the ability of metabolic and inflammatory biomarkers to predict ongoing pregnancy after IVF treatment. The study was carried out in a cohort of women presenting for natural cycle frozen embryo transfer (FET). This allowed accurate timing of peri-conceptional sampling and avoided the impact of exogenous hormones on maternal metabolic profile.

## Methods

### Patient recruitment

Women, with a regular menstrual cycle (Table 1), undergoing FET in a natural cycle with no exogenous hormones were recruited from the Assisted Conception Unit (ACU) at Glasgow Royal Infirmary between October 2007 and June 2010. Indications for IVF treatment are shown in Supplemental Table 1. At recruitment patient demographics, height, weight, waist circumference were collected and Scottish Index of Multiple Deprivation (SIMD) quintile was derived from patient postcode^12^. At day 10 after their last menstrual period (LMP) date (baseline), the women attended the ACU to commence daily hormonal sampling to detect the LH surge and time embryo (from the woman’s own oocyte) replacement. The initial pregnancy test (urinary hCG) was performed on day 18 post-LH surge with confirmation of clinical pregnancy by ultrasound on day 45 post-LH surge. Nine of the women with a negative pregnancy result at day 45 had tested positive on the hCG test at day 18 post-LH surge and were included in the non-pregnant at day 45 post-LH surge group. The study was carried out in accordance with the Declaration of Helsinki (2013). The study was approved by the Glasgow Royal Infirmary Research Ethics Committee (07/S0704/49) and all women in the study provided written informed consent.

**Table 1:** Demographic characteristics and univariate logistic regression models for prediction of ongoing pregnancy at day 45 post-LH surge.

Characteristic	Pregnant (n = 36)	Non-pregnant (n = 107)	Odds ratio	CI	*P*	C statistic
Age (years)	35.0 (4.5)	34.7 (4.7)	1.02	0.94,1.10	0.69	0.51
Current smoker n (%)	4 (11%)	23 (22%)	0.46	0.15,1.44	0.18	0.55
SIMD quintile						
1 Most affluent, 5 most deprived)						
1	6 (17%)	21 (20%)	0.57	0.17,1.92	0.78	0.57
2	8 (22%)	21 (20%)	0.80	0.25,2.52		
3	8 (22%)	32 (30%)	0.50	0.16,1.52		
4	5 (14%)	15 (14%)	0.67	0.18,2.42		
5	9 (25%)	18 (17%)	referent			
Systolic blood pressure (mmHg)	116 (13)	115 (12)	1.01	0.98,1.04	0.59	0.53
Diastolic blood pressure (mmHg)	65 (7)	66 (9)	0.98	0.94,1.03	0.40	0.54
Height (cm)	165 (7.2)	164 (6.7)	1.02	0.97,1.08	0.42	0.55
Weight (kg)	71.5 (11.3)	68.4 (13.6)	1.02	0.99,1.05	0.23	0.58
BMI (kg/m^2^)	26.5 (4.1)	25.5 (4.7)	1.05	0.97,1.14	0.26	0.58
Waist (cm)	88.6 (10.6)	85.6 (11.6)	1.03	0.99,1.06	0.16	0.59
Menstrual period length (days)	4.6 (1.5)	4.8 (1.0)	0.87	0.61,1.23	0.44	0.57
Cycle length (days)	28.8 (1.74)	28.8 (2.25)	0.99	0.83,1.19	0.92	0.49
Number of previous pregnancies ≥24 weeks						
0	35 (97%)	102 (98%)			1.00*	
>0	1 (3%)	2 (2%)				
Number of previous pregnancies <24 weeks						
0	27 (75%)	73 (70%)	0.81	0.34,1.91	0.63	0.52
>0	9 (25%)	31 (30%)				
Number of embryos transferred						
1	6 (17%)	18 (18%)	0.92	0.34,2.54	0.88	0.51
>1	30 (84%)	83 (82%)				
Treatment						
IVF	13 (36%)	53 (52%)	0.52	0.23,1.14	0.10	0.58
ICSI	23 (64%)	48 (48%)				

^*^P-value from Fisher’s Exact test.

Continuous variables are presented as mean (standard deviation) and continuous variables as number (%). Odds ratios are for a 1 unit increase in a variable. BMI = body mass index, CI = confidence interval, IVF = *in vitro* fertilisation, ICSI = intra-cytoplasmic sperm injection, SIMD = Scottish Index of Multiple Deprivation. Missing data are as follows: n = 1 pregnant (smoking status, systolic blood pressure, diastolic blood pressure, height, weight, cycle length); n = 2 pregnant (weight, BMI); n = 4 pregnant (waist); n = 6 pregnant (menstrual period length); n = 1 non-pregnant (age, smoking status, weight, BMI); n = 2 non-pregnant (waist); n = 3 non-pregnant (cycle length, number of previous pregnancies ≥ 24 weeks; number of previous pregnancies <24 weeks); n = 6 non-pregnant (number of embryos transferred; treatment); n = 8 non-pregnant (systolic blood pressure, diastolic blood pressure); n = 13 non-pregnant (menstrual period length).

### Blood sampling and biochemical analyses

Baseline blood samples, after at least a 10 hour fast, were collected approximately day 10 after LMP. Date of the LH surge was identified from the daily hormonal sampling and taken as day 0 gestation (Supplemental Figure 1). Baseline samples were collected prior to, or shortly after, LH surge (mean of 3.4 days prior; range 12 days prior to 1 day post). A further non-fasting blood sample was taken on the day of the FET (mean [standard deviation] 2.8 [0.9] days post-LH surge) prior to the procedure. Plasma and erythrocytes were collected after low speed centrifugation and frozen as aliquots at −80 °C within 2 hours. Plasma samples were analysed for total cholesterol, triglyceride, HDL cholesterol, glucose, high sensitivity CRP, non-esterified fatty acids (NEFA), insulin, hCG, IL-6, PAI-1, PAI-2 and plasma chemokines (MCP-1 [CCL2], MIP1α [CCL3], MIP1β [CCL4], IL-8 [CXCL8] and eotaxin [CCL11]) as described in the Supplemental Methods. Erythrocyte and plasma fatty analysis was carried out as described in the Supplemental Methods.

### Statistical analysis

Data are reported as mean (standard deviation) for continuous measures and number (percentage) for categorical measures. Insulin, CRP, triglyceride, IL-6 and IL-8 (CXCL8) were log transformed using the natural logarithm. Missing data are indicated and statistical analysis was carried out on available data. Differences in baseline characteristics between pregnant and non-pregnant groups were tested using two sample t-tests for continuous variables and Fisher’s Exact tests for categorical variables. Prediction of successful pregnancy outcome at day 45 post-LH surge was assessed by univariate logistic regression and *P* < 0.01 was considered significant in univariate analysis to account for multiple testing. Multivariable modelling was carried out over two phases. An initial set of stepwise logistic regression models with *P*-to-enter and *P*-to-stay < 0.15 was used to select variables from each of three variable subgroups (metabolic and inflammatory markers; erythrocyte fatty acids; plasma fatty acids) to include in the final stepwise multivariable models. Final stepwise logistic models with *P*-to-enter < 0.15 and *P*-to-stay < 0.05 were carried out using the variables selected from the initial models. Results are reported as odds ratio (OR) [95% confidence interval (CI)], Chi square score and associated *P*-value and the C-statistic for the area under the curve. Odds ratios represent an increase of 1 unit for continuous variables unless otherwise stated. *A priori* power calculations indicated at least 80% power at the 5% significant level to detect 0.47 fold differences in the majority of parameters tested as a single comparison between successful and failed pregnancy groups with n = 36 pregnancies (Supplemental Methods and Supplemental Table 2). All analyses were performed using the statistical software SAS (Version 9.3, SAS Institute, Cary, NC, USA).

### Data availability

Data may be available on request, subject to ethical approval for the specific analysis requested, DOI http://dx.doi.org/10.5525/gla.researchdata.401.

## Results

### Patient characteristics

A total of 196 FET cycles were started in the study, of which 161 were completed. For the purposes of the current analysis, repeat attempts within the study were excluded. There were 143 unique first attempts within the study period from which there were 107 unsuccessful pregnancies and 36 ongoing pregnancies defined by positive fetal heartbeat on ultrasound scan at day 45 post-LH surge (Supplemental Figure 2). Women taking part in the study were on average 35 years of age and had normal blood pressure (Table 1). Women who became pregnant did not differ from those who did not in terms of baseline age, smoking habit, obesity as measured by BMI or waist circumference, or socio-economic status. There were no differences in their previous fertility history. There were no differences between groups in the proportion of women undergoing IVF or intra-cytoplasmic sperm injection (ICSI), nor in the proportions of women having one or more than one embryo transferred.

### Univariate models for prediction of ongoing pregnancy by baseline demographics and biomarkers

None of the recorded demographic variables predicted ongoing pregnancy at day 45 post-LH surge (Table 1). Baseline erythrocyte unsaturated and saturated fatty acid concentrations were highly correlated with each other (Supplemental Figure 3). When either saturated or unsaturated erythrocyte fatty acids, or their sum (total fatty acid concentration), were individually included in a univariate logistic regression model, none predicted ongoing pregnancy (Table 2). However, if saturated and unsaturated fatty acids were entered simultaneously into a multivariable model, each was a highly significant predictor (Combined model 1, Table 2). This suggests that saturated (positive direction) and unsaturated (negative direction) fatty acids have significant individual effects that are dependent on each other. To establish the best way to capture this information, a number of univariate and multivariable models incorporating the total of saturated and unsaturated fatty acids, their difference and their ratio were carried out (Table 2). The model with the lowest Akaike Information Criterion (97.4) was the ratio of saturated to unsaturated fatty acids alone and this measure was used in all univariate and multivariate models. Analysis of baseline plasma, day of FET erythrocyte and day of FET plasma saturated and unsaturated fatty acids yielded similar results (data not shown) and their ratios were similarly used in logistic regression analysis.

**Table 2:** Logistic regression models incorporating measures of baseline erythrocyte saturated and unsaturated fatty acids for prediction of ongoing pregnancy at day 45 post LH-surge.

Variable	Odds ratio	CI	*P*	C statistic	AIC
*Univariate models*					
Saturated fatty acid (nmol/mL)	1.00	1.00, 1.00	0.89	0.51	112.5
Unsaturated fatty acid (nmol/mL)	1.00	1.00, 1.00	0.30	0.57	111.4
Total fatty acid (nmol/mL)	1.00	1.00, 1.00	0.51	0.54	112.0
Saturated/unsaturated fatty acid ratio (0.05 units)	7.19	2.33, 22.2	0.00059	0.75	97.4
Saturated – unsaturated fatty acid difference (nmol/mL)	1.02	1.00, 1.03	0.0085	0.67	104.4
*Combined model 1*				0.75	97.6
Saturated fatty acid (nmol/mL)	1.05	1.02, 1.07	0.00057		
Unsaturated fatty acid (nmol/mL)	0.97	0.95, 0.99	0.00042		
*Combined model 2*				0.75	97.6
Total fatty acid (nmol/mL)	1.01	1.00, 1.01	0.0063		
Saturated – unsaturated fatty acid difference (nmol/mL)	1.04	1.02, 1.06	0.00047		
*Combined model 3*				0.75	99.3
Total fatty acids (nmol/mL)	1.00	1.00, 1.00	0.70		
Saturated/unsaturated fatty acid ratio (0.05 units)	7.63	2.36, 24.6	0.00068		

Odds ratios are for a 1 unit increase in a variable. AIC = Akaike Information Criterion, CI = confidence interval. Plasma and erythrocyte fatty acid data was missing for n = 1 non-pregnant participant.

Baseline concentrations of plasma metabolic and inflammatory biomarkers (Table 3, Supplemental Table 3), erythrocyte fatty acids and summary measures (Table 3, Supplemental Tables 4 and 5) and plasma fatty acids and summary measures (Table 3, Supplemental Tables 6 and 7) are shown. Univariate analysis was carried out and variables that predicted ongoing pregnancy with *P* < 0.15 are shown in Table 3. These variables were included in multivariable analysis. On univariate analysis only baseline erythrocyte saturated to unsaturated fatty acid ratio predicted ongoing success at day 45 post-LH surge (*P* < 0.001) (Supplemental Figure 4).

**Table 3:** Baseline variable concentrations and univariate logistic regression models for prediction of ongoing pregnancy at day 45 post-LH surge where *P* < 0.15.

Variable	Pregnant (n = 36)	Non-pregnant (n = 107)	Odds Ratio	Confidence Interval	*P*	C-statistic
*Plasma metabolic and inflammatory markers*						
Triglyceride* (mmol/L)	1.01 (1.47)	0.90 (1.48)	2.15	(0.82, 5.63)	0.12	0.58
IL-6* (pg/mL)	1.14 (2.01)	0.90 (1.76)	1.86	(0.93, 3.72)	0.079	0.61
*Erythrocyte fatty acids and summary measures*						
14:0 (nmol/mL)	12.8 (7.06)	9.60 (6.35)	1.07	1.00, 1.15	0.040	0.67
17:0 (nmol/mL)	6.94 (3.06)	5.72 (2.04)	1.21	1.00, 1.45	0.045	0.60
18:1 n-7 (nmol/mL)	6.31 (8.89)	10.64 (10.98)	0.96	0.92, 1.00	0.065	0.63
20:1 n-9 (nmol/mL)	4.24 (1.06)	4.94 (1.35)	0.61	0.41, 0.92	0.019	0.65
24:1 n-9 (nmol/mL)	64.5 (9.8)	70.2 (12.6)	0.96	0.92, 1.00	0.036	0.65
20:2 n-6 (nmol/mL)	0.66 (1.24)	1.30 (1.77)	0.76	0.55, 1.03	0.078	0.59
22:4 n-6 (nmol/mL)	38.5 (6.7)	43.1 (10.3)	0.94	0.89, 1.00	0.032	0.64
18:3 n-3 (nmol/mL)	3.93 (2.08)	2.95 (2.22)	1.24	1.00, 1.54	0.051	0.63
% Total n-6	27.8 (1.27)	28.4 (1.3)	0.67	0.46, 0.98	0.039	0.62
Unsaturated Index	150.1 (4.1)	151.5 (4.0)	0.92	0.82, 1.03	0.14	0.57
Average chain length^†^ (0.05 units)	18.66 (0.05)	18.69 (0.06)	0.63	0.42, 0.95	0.027	0.66
Saturated/unsaturated ratio^†^ (0.05 units)	0.78 (0.02)	0.76 (0.02)	7.19	2.33, 22.17	0.00059	0.75
*Plasma fatty acids and summary measures*						
14:0 (nmol/mL)	118 (51)	98.3 (45.8)	1.01	1.00, 1.02	0.079	0.62
17:0 (nmol/mL)	30.0 (10.1)	25.6 (9.8)	1.05	1.00, 1.10	0.059	0.61
18:0 (nmol/mL)	618 (117)	577 (98)	1.00	1.00, 1.01	0.11	0.60
14:1 n-7 (nmol/mL)	41.7 (35.1)	29.6 (29.1)	1.01	1.00, 1.03	0.10	0.59
24:1 n-9 (nmol/mL)	88.5 (10.4)	94.3 (15.9)	0.97	0.94, 1.00	0.072	0.62
Saturated/unsaturated ratio^†^ (0.05 units)	0.52 (0.05)	0.49 (0.04)	2.12	1.20, 3.76	0.010	0.66
% Total n-6	37.4 (3.09)	38.5 (3.4)	0.90	0.79, 1.04	0.15	0.60
Average chain length^†^ (0.05 units)	17.80 (0.08)	17.84 (0.09)	0.75	0.57, 1.00	0.047	0.65

Continuous variables are presented as mean (standard deviation) or * geometric means (standard deviation). Odds ratios are for a 1 unit increase in a variable apart from^†^. IL-6 = interleukin 6. IL-6 data was missing for n = 1 pregnant and n = 50 non-pregnant participants. Plasma triglyceride and plasma and erythrocyte fatty acid data was missing for n = 1 non-pregnant participant.

### Univariate models for prediction of successful pregnancy outcome by day of FET, non-fasting biomarkers

Non-fasting blood samples were taken just prior to the FET procedure on median day 3 post-LH surge and metabolic and inflammatory biomarkers assessed as well as erythrocyte and plasma fatty acids (data not shown). Day of FET non-fasting plasma log-triglyceride (3.48 [1.43, 8.48], *P* = 0.006, C-statistic 0.66) was associated with a higher odds of ongoing pregnancy and additionally insulin, CRP and NEFA (all *P* < 0.15) were included in stepwise multivariable modelling. Erythrocyte 14:0 (1.09 [1.02, 1.16], *P* = 0.0087, C-statistic 0.69), n-6 polyunsaturated fatty acids (PUFA) (0.56 [0.36, 0.86], *P* = 0.0076, C-statistic 0.68), average chain length (0.51, [0.33, 0.76], *P* = 0.0012, C-statistic 0.73) and saturated/unsaturated fatty acid ratio (0.05 units) (6.70, [2.25, 19.88], *P* = 0.0062, C-statistic 0.73) were associated with ongoing pregnancy. Additionally erythrocyte 14:1 n-7, 18:1 n-7, 18:3 n-3, 20:1 n-9, 20:3 n-6, 20:4 n-6, 22:4 n-6, 22:5 n-3, 24:1 n-9, unsaturated index and % C20–22 fatty acids (all *P* < 0.15) were included in stepwise multivariable modelling. Plasma 14:0 (1.01 [1.00, 1.02], *P* = 0.008, C-statistic 0.66), 17:0 (1.07 [1.02, 1.12], *P* = 0.007, C-statistic 0.68), plasma saturated/unsaturated fatty acid ratio (0.05 units) (2.31 [1.30, 4.09], *P* = 0.0041, C-statistic 0.69), n-6 PUFA (0.80 [0.68, 0.93] *P* = 0.0049, C-statistic 0.70), unsaturated index (0.91 [0.84, 0.98], *P* = 0.0086, C-statistic 0.68) and fatty acid average chain length (0.05 units) (0.65 [0.48, 0.88], *P* = 0.0046, C-statistic 0.70) were associated with ongoing pregnancy. Plasma 12:0, 14:1 n-7, 16:0, 16:1 n-7; 18:0, 18:1 n-9, 20:1 n-9, 24:1 n-9 and % C20–22 fatty acids (all *P* < 0.15) were also included in stepwise multivariable modelling.

### Multivariable models for prediction of ongoing pregnancy

Initial stepwise multivariable models (*P*-to-enter, *P*-to-stay < 0.15) were carried out using the baseline and day of FET variables, selected at *P* < 0.15 on univariate analysis (Table 3 for baseline measures and listed above for day of FET measures). This resulted in the selection of log triglyceride, log IL-6, log insulin, erythrocyte saturated to unsaturated fatty acid ratio, average chain length, 18:3 n-3, 20:1 n-9 and 24:1 n-9; and plasma saturated to unsaturated fatty acid ratio, 17:0 and 24:1 n-9 for inclusion in the final stepwise models.

Table 4 shows the final multivariable logistic regression models for predictors of ongoing pregnancy. The only baseline predictor of ongoing pregnancy remaining after stepwise multivariable analysis (*P*-to-enter < 0.15, *P*-to-stay < 0.05) was saturated to unsaturated fatty acid ratio with an odds ratio of 7.19 (2.33, 22.17) per 0.05 unit increase, *P* = 0.0006. The C-statistic for this prediction model was 0.75.

**Table 4:** Multivariable logistic regression models for predicting pregnancy success at 45 days post-LH surge.

Visit/Covariate	Multivariable		
	Odds Ratio (95% confidence interval)	*P*-value	C-statistic (Area Under Curve)
*Baseline* (*mean of 3.4 days prior to LH surge*)			
Erythrocyte saturated/unsaturated fatty acid ratio (0.05 units)	7.19 (2.33, 22.17)	0.0006	0.75
*Day of FET* (*mean of 2.8 days post-LH surge*)			
Erythrocyte saturated/unsaturated fatty acid ratio (0.05 units)	7.11 (2.05, 24.71)	0.002	0.84
Erythrocyte fatty acid average chain length (0.05 units)	0.58 (0.37, 0.92)	0.020	
Log [triglyceride (mmol/L)]	4.23 (1.05, 16.95)	0.042	

Covariates to enter the stepwise logistic regression were initially selected using baseline, or day of FET (mean 2.8 days post-LH surge) stepwise logistic regression models run for all covariates (plasma and erythrocyte metabolic, inflammatory and fatty acid markers) *P*-to-enter and *P*-to-stay < 0.15). Variables included were baseline or day of FET triglyceride, IL-6 and insulin; erythrocyte 18:3 n-3, 20:1 n-9, 24:1 n-9, saturated/unsaturated fatty acid ratio and average chain length; and plasma 17:0, 24:1 n-9 and saturated/unsaturated fatty acid ratio. The final multivariable model is a stepwise model with *P*-to-enter and *P*-to-stay < 0.05. Data shown are the odds ratio (for the given unit of change), and the 95% confidence interval, with the associated Chi square score, *P*-value, and the C-statistic (for the Area under the Curve). FET = frozen embryo transfer.

When non-fasted day of FET covariates were used to predict ongoing pregnancy, erythrocyte saturated to unsaturated fatty acid ratio OR 7.11 (2.05, 24.71) per 0.05 unit increase, *P* = 0.002, erythrocyte fatty acid average chain length OR 0.58 (0.37, 0.92) per 0.05 unit increase, *P* = 0.020 and plasma log-triglycerides OR 4.23 (1.05, 16.95) *P* = 0.042 predicted ongoing pregnancy. The C-statistic for the prediction model based on non-fasting day of FET covariates was 0.84.

## Discussion

In women undergoing natural cycle FET, the erythrocyte ratio of saturated to unsaturated fatty acids prior to pregnancy predicted ongoing pregnancy at day 45 post-LH surge. Similarly, on day of FET, higher erythrocyte saturated to unsaturated fatty acid ratio and shorter average chain length, in addition to higher concentrations of plasma triglycerides, predicted ongoing pregnancy. Erythrocytes have a lifespan of 120 days, though their fatty acid composition may reflect more recent changes in concentration^13^. As erythrocyte composition reflects that of tissue stores^14^, this suggests that the quality of stored fatty acids has an impact on pregnancy outcome. A higher ratio of saturated to unsaturated fatty acids in the diet is commonly linked to metabolic diseases such as coronary heart disease, however this issue is complex and there is a lack of consensus on the scientific evidence for these associations^15,16^. Erythrocyte fatty acid composition is unaffected by fasting status, thus it is reassuring to see similar erythrocyte predictors at both time points. The prediction of ongoing pregnancy by non-fasting plasma triglyceride levels on day of FET, which are increased post-prandially, suggests that pre-implantation maternal handling of dietary lipids may be important in the preparation for pregnancy.

Serum fatty acids have previously been linked to pregnancy outcome in an IVF population. Higher serum α-linolenic acid concentrations collected on day of oocyte retrieval were associated with a lower chance of pregnancy, although this may not be independent of endometriosis^6^. These data were not replicated in a subsequent study which instead found a high linoleic acid to α-linolenic acid ratio was associated with pregnancy success after adjustment for confounders^17^. Taken together with our study, these preliminary data indicate that the proportions of plasma fatty acids of different functionality may be an important factor in pregnancy success.

Maternal metabolic status could influence fertility by improving oocyte/embryo quality or endometrial receptivity. The mammalian blastocyst is dependent on its own energy stores to drive cell division^18^ and endogenous triglycerides are a key energy source during oocyte maturation and preimplantation embryo development^19^. Mouse models suggest that fatty acid oxidation is critical for oocyte function, embryo development, implantation and endometrial stromal cell decidualisation^20,21^. In human oocytes 80% of fatty acids are saturated^22,23^. Exposure of the blastocyst to external lipid appears to be cytotoxic^24^ which suggests that fatty acid supply via uterine secretions may not occur. Human embryos that successfully develop to blastocysts have lower triglyceride content, although it is difficult to determine if this is due to less initial storage or greater oxidation rates providing more energy while the blastocyst was being formed^2^. A well-stocked oocyte in terms of lipid available for oxidation (i.e. saturated fat) may have an advantage over one that lacks such lipid.

The oocytes in the current study were retrieved at least three months prior to FET so our baseline biomarkers do not necessarily reflect the environment of oocyte development but will reflect the implantation environment. Uterine receptivity is affected by changes in metabolic environment associated with maternal obesity or polycystic ovarian syndrome (PCOS) (reviewed in^25^). Pregnancy hormones influenced adipokine secretion from and increased lipolysis in an adipocyte cell line^26^. The pregnancy hormone-conditioned medium from these adipocytes increased receptivity marker expression in an endometrial cell line^26^. Adipocytes contain predominately saturated fatty acids whose composition reflects that of plasma fatty acids which in turn are influenced by dietary intake^27^. A significant increase in fatty acid release into plasma from adipocytes may lead to an increase in the saturated to unsaturated fatty acid ratio. Weight loss in overweight women with PCOS increased endometrial expression of genes involved in glucose homeostasis, which may improve endometrial receptivity^28^. Improvements in insulin sensitivity at the endometrium may also enhance tissue fatty acid uptake and oxidation.

The analysis of non-fasting biomarkers taken immediately prior to FET produced a predictive model with a higher C statistic than that using fasting biomarkers pre-LH surge. Erythrocyte fatty acid average chain length appeared as a negative predictor reinforcing the suggestion that shorter chain fatty acids commonly used in energy storage are important for pregnancy success. It is interesting that higher plasma triglyceride concentrations were predictive of an ongoing pregnancy in the non-fasting biomarker analysis. Plasma triglyceride concentrations under these conditions represent recent post prandial intake of lipid. This might suggest that women with higher plasma levels of dietary lipids may be have better endometrial receptivity or improved oocyte quality. A recent report that higher dietary dairy intake was associated with increased live birth rate in an IVF population, at least in older women, may support this concept^29^. FET of autologous or donor embryos comprises 37.7% of assisted conception cycles currently undertaken in the United Sates (CDC Assisted Reproductive Technology Annual Report 2015). Biomarkers, which can indicate the potential for the success of different assisted conception protocols, are helpful both in tailoring treatment to the individual patients and in devising intervention strategies, e.g. dietary supplementation or intervention, that may improve success rates in assisted conception treatment.

One strength of our study was the prospective design in an IVF population allowing accurate peri-conceptional timing of sampling. All women had a natural menstrual cycle and results were free from the impact of exogenous hormones. Use of frozen, rather than fresh, embryos may mean our results are specific to frozen embryos. We did not observe the expected associations between maternal BMI, smoking or number of embryos transferred^30^ and ongoing pregnancy probably due to the small number of pregnancies and the limited BMI range within our population limiting statistical power. Pregnancy success rates in this study were low reflecting day 3 embryo transfers and the absence of pre-implantation genetic screening. Nine women who tested positive for pregnancy on hCG test at day 18 post-LH surge, but negative at day 45 post-LH surge ultrasound scan were included as unsuccessful pregnancies. These women may represent a particular subgroup where implantation is successful, but pregnancy maintenance is not. This group was too small to be analysed separately and as our *a priori* pregnancy outcome was positive fetal heartbeat at day 45 post-LH surge we included them in our unsuccessful pregnancy group.

We cannot determine whether the relationships we observed only occur under conditions of compromised fertility or extend to natural conception in the wider population. The peri-conceptional period is a key opportunity for interventional strategies that is routinely missed due to management of most pregnancies commencing at antenatal booking around 10–15 weeks of gestation. Evidence-based strategies, delivered either in the preconception or antenatal periods, to reduce adverse pregnancy outcomes are being sought. If our results are validated, the saturated to unsaturated fat ratio in erythrocytes could be explored as a marker of maternal metabolic health. However it should be noted that the absolute difference in saturated to unsaturated fatty acid ratio between groups was very small and while it may suggest potential biological metabolic pathways of importance in conception it is unlikely to be a good biomarker in itself.

In summary, higher peri-conceptional saturated to unsaturated fatty acid ratio predicted ongoing pregnancy after natural cycle FET and may reflect a maternal nutritional status that facilitates pregnancy success in this assisted conception scenario. Dietary interventions could be used to manipulate the saturated to unsaturated fatty acid ratio and better assess its effect on pregnancy outcome both in assisted conception and free-living populations.

## Electronic supplementary material

Supplementary Information
