# Supplementary material for: Pre-conception maternal erythrocyte saturated to unsaturated fatty acid ratio predicts pregnancy after natural cycle frozen embryo transfer
Source: Sci Rep. 2018 Jan 19;8:1216. doi: 10.1038/s41598-018-19500-0 (PMC5775249; doi:10.1038/s41598-018-19500-0)
Supplement: Supplementary file 1 — Supplementary Information [file 41598_2018_19500_MOESM1_ESM.doc]

**Pre-conception maternal erythrocyte saturated to unsaturated fatty acid ratio predicts pregnancy after natural cycle frozen embryo transfer.**

Christopher C. Onyiaodike^1^, Heather M. Murray^2^, Ruiqi Zhang^2^, Barbara J. Meyer^3^, Fiona Jordan^4^, E. Ann Brown^1^, Robert J. B. Nibbs^5^, Helen Lyall^1^, Naveed Sattar^4^, Scott M. Nelson^1^, Dilys J. Freeman^4^.

^1^School of Medicine, University of Glasgow, Glasgow, UK;

^2^Robertson Centre for Biostatistics, University of Glasgow, Glasgow, UK;

^3^School of Medicine, Metabolic Research Centre, University of Wollongong, Wollongong, NSW, Australia;

^4^Institute of Cardiovascular and Medical Sciences, University of Glasgow, Glasgow, UK;

^5^Institute of Infection, Immunity and Inflammation, University of Glasgow, Glasgow, UK.

**Supplemental Information**

**Supplemental Methods**

*Patients*

**Supplemental Table 1. Indications for *in vitro* fertilisation in study participants.** PCOS – polycystic ovarian syndrome, PGD = pre-implantation genetic diagnosis

| Characteristic | Pregnant  (n=36) | Non-Pregnant  (n=107) |
| --- | --- | --- |
| Chemotherapy | 0 (0.0%) | 1 (0.9%) |
| Endometriosis | 5 (13.9%) | 13 (12.2%) |
| Genetic factor | 0 (0%) | 1 (0.9%) |
| Male factor | 18 (50.0%) | 47 (43.9) |
| PCOS | 1 (2.8%) | 3 (2.8%) |
| Pelvic adhesion | 1 (2.8%) | 0 (0%) |
| PGD | 1 (2.8%) | 0 (0%) |
| Tubal | 7 (19.4%) | 33 (30.8%) |
| Other/unexplained | 6 (16.7%) | 19 (17.8%) |
| Missing | 0 (0%) | 1 (0.9%) |
| Twin pregnancy | 9 (25.0%) | - |

*Plasma biochemical analyses*

Total cholesterol (sensitivity 0.35 mmol/L), triglyceride (0.26mmol/L) and HDL cholesterol (0.08mmol/L)^1^, glucose (0.11 mmol/L) and high sensitivity CRP (0.48mmol/L) assays^2^ were performed by Vascular Biochemistry, University of Glasgow. Plasma non-esterified fatty acids (NEFA, sensitivity 0.1 mmol/L) were quantitated by colorimetric assay (Wako, Alpha Laboratories, Eastleigh, UK). Insulin (sensitivity 0.07mU/L) quantitation was performed by ELISA (Mercodia, Sweden). hCG (sensitivity 1.1 IU/L) measured on an Immulite autoanalyser (Siemens/DPC, Erlangen Germany). Plasma IL-6 (sensitivity 0.039pg/mL) (R&D Systems, Abingdon, UK), PAI-1 (0.5ng/mL) (TriniLIZE, Stago BNL, Leiden, The Netherlands) and PAI-2 (50pg/mL) (American Diagnostica Inc, Invitech, Huntingdon, UK) were measured using commercial ELISA. Plasma chemokines (MCP-1 [CCL2, sensitivity 0.9 pg/mL], MIP1α [CCL3, 3.5 pg/mL], MIP1β [CCL4, 4.5 pg/mL], IL-8 [CXCL8, 0.2 pg/mL] and eotaxin [CCL11, 1.2 pg/mL]) were assayed on a BioPlex Suspension Array System (BioRad, Hemel Hempstead, UK) using a commercial kit (Millipore, Watford, UK). All assays were performed according to the manufacturers’ instructions.

*Erythrocyte and plasma fatty acid analysis*

Erythrocytes (400 μL packed), collected after removal of plasma by centrifugation, were resuspended in TRIS buffer (10mM Bis Tris, 2mM Na_2_EDTA, pH 7.2) at room temperature for 30 minutes and then ultracentrifuged (30 mins, 49,000rpm, 4^o^C, Beckman, USA) to pellet the erythrocyte membranes. The supernatant was discarded and the erythrocyte membrane pellet was resuspended in 200μL distilled water. Plasma samples were thawed and prepared for fatty acid analysis according to Swierk et al^3^. The direct transesterification procedure and gas chromatography of derivatised fatty acids was carried out as previously described^4^.

*Power Calculation*

Power calculations are provided in Supplemental Table 2. In the absence of specific pilot data relating to differences in maternal metabolic parameters between successful and unsuccessful pregnancy, first trimester data derived from a longitudinal study of late gestations was examined^5^. It was assumed that the difference in a metabolic parameter that might be related to a failed rather than a successful pregnancy would be similar to the mean difference observed between lean (BMI tertile 1) and obese (BMI tertile 3) mothers at 12 weeks’ gestation (data in this format unpublished). Using this pilot data an estimate of 28 successful pregnancies for 80% power and 37 pregnancies for 90% power was sufficient to identify this magnitude of difference between failed and successful pregnancy groups. Thus there is at least 80% power at the 5% significant level to detect differences in the majority of parameters tested between successful and failed pregnancy groups with n=36 pregnancies.

**Supplemental Table 2. Power Calculations**

| Parameter | Delta (mean BMI tertile 3- BMI tertile 1 at 1^st^ trimester) | Standardised delta | Standardised  Sigma | Power 80% (90%) (n) BMI tertile 3- BMI tertile 1 difference  (n) |
| --- | --- | --- | --- | --- |
| HOMA | 8.18 | 2.35 | 1.01 | 5 (6) |
| Fasting TG | 0.53 mM | 0.47 | 0.27 | 7 (9) |
| CRP | 8.04 mg/L | 2.10 | 0.78 | 4 (5) |
| PAI-2 | -26 ng/mL | 0.56 | 0.73 | 28 (37) |

1 Third Report of the National Cholesterol Education Program (NCEP) Expert Panel on Detection, Evaluation, and Treatment of High Blood Cholesterol in Adults (Adult Treatment Panel III) final report. *Circulation* **106**, 3143-3421 (2002).

2 Packard, C. J. *et al.* Lipoprotein-associated phospholipase A2 as an independent predictor of coronary heart disease. West of Scotland Coronary Prevention Study Group. *The New England journal of medicine* **343**, 1148-1155, doi:10.1056/nejm200010193431603 (2000).

3 Swierk, M., Williams, P. G., Wilcox, J., Russell, K. G. & Meyer, B. J. Validation of an Australian electronic food frequency questionnaire to measure polyunsaturated fatty acid intake. *Nutrition (Burbank, Los Angeles County, Calif.)* **27**, 641-646, doi:10.1016/j.nut.2010.06.011 (2011).

4 Stewart, F. *et al.* Longitudinal assessment of erythrocyte fatty acid composition throughout pregnancy and post partum. *Lipids* **42**, 335-344, doi:10.1007/s11745-006-3005-5 (2007).

5 Stewart, F. M. *et al.* Longitudinal assessment of maternal endothelial function and markers of inflammation and placental function throughout pregnancy in lean and obese mothers. *The Journal of clinical endocrinology and metabolism* **92**, 969-975, doi:10.1210/jc.2006-2083 (2007).

**Supplemental Results**

**Supplemental Table 3. Baseline plasma biomarkers with *P*>0.15 and univariate logistic regression models for prediction of ongoing pregnancy at day 45 post-LH surge. Continuous variables are presented as mean (standard deviation) or * geometric means (standard deviation). Odds ratios are for a 1 unit increase in a variable. CRP = C-reactive protein, hCG = human chorionic gonadotrophin, HDL = high density lipoprotein, IL-6 = interleukin 6, IL-8 = interleukin 8, MCP = monocyte chemoattractant protein, MIP = macrophage inflammatory protein, NEFA = non-esterified fatty acids, PAI = plasminogen activator inhibitor. The following data were missing: n=1 pregnant (insulin, IL-6, IL-8, PAI-1, PAI-2, eotaxin, MCP-1, MIP-1α, MIP-1β); n=2 pregnant (hCG); n=13 pregnant (NEFA); n=1 non-pregnant (total cholesterol, HDL cholesterol, triglyceride, CRP); n=3 non-pregnant (glucose); n=39 non-pregnant (insulin); n=50 non-pregnant (IL-6); n=51 non-pregnant for (IL-8, eotaxin, MCP-1, MIP-1α, MIP-1β); n=54 non-pregnant (hCG, PAI2); n=56 non-pregnant (PAI-1); n=70 non-pregnant NEFA.**

| Biomarker | Pregnant (n=36) | Non-pregnant (n=107) | Odds Ratio | Confidence Interval | *P* | C-statistic |
| --- | --- | --- | --- | --- | --- | --- |
| hCG  (IU/L) | 0.04 (0.21) | 0.08 (0.34) | 0.54 | (0.09, 3.15) | 0.49 | 0.51 |
| Total cholesterol (mmol/L) | 4.72 (0.72) | 4.81 (0.76) | 0.85 | (0.51, 1.41) | 0.52 | 0.55 |
| HDL cholesterol (mmol/L) | 1.50 (0.30) | 1.59 (0.42) | 0.54 | (0.20, 1.51) | 0.24 | 0.54 |
| NEFA  (mmol/L) | 0.25 (0.15) | 0.27 (0.14) | 0.52 | (0.01, 22.7) | 0.73 | 0.53 |
| Glucose  (mmol/L) | 4.80 (0.46) | 4.79 (0.75) | 1.03 | (0.59, 1.80) | 0.91 | 0.52 |
| Insulin*  (mU/L) | 6.17 (2.42) | 6.89 (1.91) | 0.81 | (0.46, 1.43) | 0.47 | 0.54 |
| CRP*  (mg/L) | 1.26 (2.97) | 0.94 (2.94) | 1.27 | (0.90, 1.80) | 0.17 | 0.58 |
| IL-6  (pg/mL) | 1.14 (2.01) | 0.90 (1.76) | 1.86 | (0.93 , 3.72) | 0.079 | 0.61 |
| IL-8* (CXCL8)  (pg/mL) | 17.0 (3.8) | 19.7 (4.7) | 0.93 | (0.70, 1.25) | 0.64 | 0.52 |
| PAI-1  (ng/mL) | 27.0 (25.7) | 20.4 (12.3) | 1.02 | (0.99, 1.05) | 0.16 | 0.60 |
| PAI-2  (ng/mL) | 1.29 (0.52) | 1.17 (1.47) | 1.09 | (0.76, 1.57) | 0.65 | 0.65 |
| Eotaxin (CCL11)  (pg/mL) | 125 (72) | 139 (119) | 1.00 | (0.99, 1.00) | 0.51 | 0.48 |
| MCP-1 (CCL2)  (pg/mL) | 263 (109) | 271 (165) | 1.00 | (1.00, 1.00) | 0.79 | 0.46 |
| MIP-1α (CCL3)  (pg/mL) | 84.1 (95.4) | 98.8 (106.0) | 1.00 | (0.99, 1.00) | 0.51 | 0.56 |
| MIP-1β (CCL4)  (pg/mL) | 226 (290) | 269 (356) | 1.00 | (1.00, 1.00) | 0.55 | 0.54 |

**Supplemental Table 4.** **Baseline erythrocyte fatty acid concentrations with *P*>0.15 and univariate logistic regression models for prediction of ongoing pregnancy at day 45 post-LH surge.** Continuous variables are presented as mean (standard deviation). Odds ratios are for a 1 unit increase in a variable. CI = confidence interval, MUFA = monounsaturated fatty acid, PUFA = polyunsaturated fatty acid, SAFA = saturated fatty acid. Erythrocyte fatty acid data was missing for n=1 non-pregnant participant.

| Fatty Acid (nmol/mL) | Pregnant (n=36) | Non-pregnant (n=107) | Odds ratio | CI | *P* | C statistic |
| --- | --- | --- | --- | --- | --- | --- |
| SAFA |  |  |  |  |  |  |
| 12:0 | 0.95 (2.79) | 0.03 (0.17) | 2.41 | 0.49, 11.9 | 0.28 | 0.56 |
| 16:0 | 361 (47) | 368 (55) | 1.00 | 0.99, 1.01 | 0.61 | 0.53 |
| 18:0 | 250 (36) | 253 (36) | 1.00 | 0.99, 1.01 | 0.63 | 0.54 |
| 20:0 | 7.70 (2.03) | 7.51 (1.61) | 1.06 | 0.83, 1.36 | 0.65 | 0.52 |
| 22:0 | 24.5 (4.9) | 24.1 (4.0) | 1.02 | 0.93, 1.13 | 0.64 | 0.51 |
| 24:0 | 54.9 (8.5) | 54.1 (9.6) | 1.01 | 0.96, 1.06 | 0.68 | 0.53 |
| MUFA |  |  |  |  |  |  |
| 14:1 n-7 | 0.67 (2.08) | 0.12 (0.79) | 1.33 | 0.90, 1.97 | 0.16 | 0.55 |
| 16:1 n-7 | 13.2 (12.7) | 13.1 (11.4) | 1.00 | 0.96, 1.04 | 0.97 | 0.45 |
| 17:1 n-7 | 63.7 (10.3) | 62.7 (9.5) | 1.01 | 0.97, 1.06 | 0.65 | 0.52 |
| 18:1 n-9 | 199 (33) | 203 (35) | 1.00 | 0.98, 1.01 | 0.61 | 0.53 |
| PUFA n-6 |  |  |  |  |  |  |
| 18:2 n-6 | 151 (23) | 155 (29) | 1.00 | 0.98, 1.01 | 0.55 | 0.54 |
| 18:3 n-6 | 1.34 (1.27) | 1.16 (1.18) | 1.13 | 0.78, 1.63 | 0.53 | 0.53 |
| 20:3 n-6 | 26.5 (7.3) | 27.5 (6.0) | 0.98 | 0.91, 1.05 | 0.48 | 0.55 |
| 20:4 n-6 | 228 (32) | 240 (42) | 0.99 | 0.98, 1.00 | 0.16 | 0.59 |
| 22:5 n-6 | 6.41 (1.61) | 6.72 (1.98) | 0.91 | 0.71, 1.17 | 0.47 | 0.53 |
| PUFA n-3 |  |  |  |  |  |  |
| 20:5 n-3 | 13.1 (5.1) | 11.7 (4.7) | 1.06 | 0.97, 1.17 | 0.20 | 0.58 |
| 22:5 n-3 | 35.8 (6.3) | 37.5 (7.4) | 0.96 | 0.90, 1.03 | 0.28 | 0.56 |
| 22:6 n-3 | 60.3 (16.3) | 58.1 (13.7) | 1.01 | 0.98, 1.04 | 0.50 | 0.55 |

**Supplemental Table 5.** **Baseline erythrocyte fatty acid summary measures and univariate logistic regression models for prediction of successful pregnancy at day 45 post-LH surge.** Continuous variables are presented as mean (standard deviation). Odds ratios are for a 1 unit increase in a variable. CI = confidence interval, MUFA = monounsaturated fatty acid, PUFA = polyunsaturated fatty acid. Erythrocyte fatty acid data was missing for n=1 non-pregnant participant.

| Summary measure | Pregnant (n=36) | Non-pregnant (n=107) | Odds ratio | CI | *P* | C statistic |
| --- | --- | --- | --- | --- | --- | --- |
| % MUFA | 21.4 (0.8) | 21.8 (1.0) | 0.69 | 0.42, 1.13 | 0.14 | 0.60 |
| % PUFA | 34.6 (0.8) | 34.9 (0.9) | 0.60 | 0.34, 1.04 | 0.070 | 0.60 |
| % Total n-9 | 16.3 (1.0) | 16.6 (0.9) | 0.76 | 0.47, 1.22 | 0.25 | 0.60 |
| % Total n-7 | 5.12 (0.81) | 5.18 (0.78) | 0.91 | 0.51, 1.60 | 0.73 | 0.52 |
| % Total n-3 | 6.89 (1.04) | 6.62 (1.02) | 1.30 | 0.84, 2.03 | 0.24 | 0.60 |
| n-6/n-3 ratio | 4.16 (0.85) | 4.41 (0.83) | 0.68 | 0.38, 1.19 | 0.18 | 0.62 |
| C20-22 | 30.71 (1.12) | 31.01 (1.08) | 0.78 | 0.51, 1.18 | 0.23 | 0.57 |
| 20:4n-6/20:3n-6 | 9.16 (2.50) | 8.96 (1.79) | 1.05 | 0.85, 1.29 | 0.67 | 0.50 |
| 20:3n-6/18:2n-6 | 0.17 (0.04) | 0.18 (0.03) | 0.008 | 0, 4176 | 0.47 | 0.54 |
| 18:1n-9/18:0 | 0.80 (0.06) | 0.80 (0.06) | 0.59 | 0.00, 928.9 | 0.89 | 0.54 |
| 18:0/16:0 | 0.69 (0.03) | 0.69 (0.04) | 0.23 | 0.00, 47794 | 0.81 | 0.51 |

**Supplemental Table 6.** **Baseline plasma fatty acid concentrations and univariate logistic regression models for prediction of successful pregnancy at day 45 post LH-surge.** Continuous variables are presented as mean (standard deviation). Odds ratios are for a 1 unit increase in a variable. CI = confidence interval. Plasma fatty acid data was missing for n=1 non-pregnant participant.

| Fatty Acid (nmol/mL) | Pregnant (n=36) | Non-pregnant (n=107) | Odds ratio | CI | *P* | C statistic |
| --- | --- | --- | --- | --- | --- | --- |
| SAFA |  |  |  |  |  |  |
| 12:0 | 35.6 (41.2) | 29.2 (41.8) | 1.00 | 0.99, 1.02 | 0.49 | 0.55 |
| 16:0 | 2099 (444) | 1991 (439) | 1.00 | 1.00, 1.00 | 0.28 | 0.58 |
| 20:0 | 33.6 (6.0) | 32.8 (6.7) | 1.02 | 0.95, 1.10 | 0.56 | 0.56 |
| 22:0 | 51.8 (9.8) | 52.6 (9.0) | 0.99 | 0.94, 1.04 | 0.70 | 0.52 |
| 24:0 | 40.2 (5.8) | 41.3 (7.3) | 0.98 | 0.91, 1.04 | 0.46 | 0.53 |
| MUFA |  |  |  |  |  |  |
| 16:1 n-7 | 210 (88) | 199 (98) | 1.00 | 1.00, 1.01 | 0.61 | 0.56 |
| 17:1 n-7 | 30.6 13.3) | 29.1 (14.4) | 1.01 | 0.98, 1.04 | 0.64 | 0.53 |
| 18:1 n-9 | 1760 (427) | 1736 (457) | 1.00 | 1.00, 1.00 | 0.80 | 0.53 |
| 20:1 n-9 | 17.9 (6.7) | 16.8 (8.1) | 1.02 | 0.96, 1.08 | 0.52 | 0.53 |
| PUFA n-6 |  |  |  |  |  |  |
| 18:2 n-6 | 2521 (385) | 2542 (411) | 1.00 | 1.00, 1.00 | 0.80 | 0.53 |
| 18:3 n-6 | 42.5 (20.5) | 40.4 (19.9) | 1.01 | 0.98, 1.03 | 0.64 | 0.53 |
| 20:2 n-6 | 7.2 (6.2) | 6.5 (5.9) | 1.02 | 0.95, 1.10 | 0.57 | 0.50 |
| 20:3 n-6 | 135 (39) | 134 (34) | 1.00 | 0.99, 1.01 | 0.84 | 0.49 |
| 20:4 n-6 | 546 (123) | 543 (106) | 1.00 | 1.00, 1.00 | 0.91 | 0.53 |
| 22:4 n-6 | 11.7 (4.5) | 11.7 (4.1) | 1.00 | 0.90, 1.11 | 0.99 | 0.51 |
| 22:5 n-6 | 7.2 (4.3) | 6.2 (4.7) | 1.05 | 0.95, 1.16 | 0.33 | 0.55 |
| PUFA n-3 |  |  |  |  |  |  |
| 18:3 n-3 | 64.5 (23.7) | 67.0 (26.0) | 1.00 | 0.98, 1.01 | 0.65 | 0.53 |
| 20:5 n-3 | 81.8 (44.4) | 74.1 (39.1) | 1.01 | 0.99, 1.02 | 0.42 | 0.57 |
| 22:5 n-3 | 46.8 (16.4) | 47.5 (14.2) | 1.00 | 0.97, 1.03 | 0.84 | 0.54 |
| 22:6 n-3 | 141 (50) | 130 (51) | 1.00 | 1.00, 1.01 | 0.37 | 0.59 |

**Supplemental Table 7.** **Plasma fatty acid summary measures and univariate logistic regression models for prediction of successful pregnancy at day 45 post-LH surge.** Continuous variables are presented as mean (standard deviation). Odds ratios are for a 1 unit increase in a variable. CI = confidence interval. Plasma fatty acid data was missing for n=1 non-pregnant participant.

| Summary measure | Pregnant (n=36) | Non-pregnant (n=107) | Odds ratio | CI | *P* | C statistic |
| --- | --- | --- | --- | --- | --- | --- |
| % MUFA | 24.9 (2.3) | 25.0 (3.1) | 0.98 | 0.83, 1.15 | 0.76 | 0.51 |
| % PUFA | 414.3 (3.1) | 42.3 (3.5) | 0.92 | 0.80, 1.05 | 0.20 | 0.58 |
| % Total n-9 | 21.1 (2.0) | 21.3 (2.6) | 0.96 | 0.79, 1.16 | 0.66 | 0.52 |
| % Total n-7 | 3.8 (0.8) | 3.8 (0.9) | 1.07 | 0.63, 1.80 | 0.81 | 0.53 |
| % Total n-3 | 3.78 (0.92) | 3.7 (1.0) | 1.08 | 0.68, 1.70 | 0.74 | 0.54 |
| n-6/n-3 ratio | 10.53 (3.06) | 11.01 (2.90) | 0.95 | 0.81, 1.10 | 0.47 | 0.58 |
| Unsaturated Index | 133.6 (7.1) | 135.8 (7.2) | 0.96 | 0.90, 1.02 | 0.19 | 0.58 |
| C20-22 | 12.94 (1.84) | 13.06 (1.79) | 0.96 | 0.75, 1.24 | 0.77 | 0.52 |
| 20:4n-6/20:3n-6 | 4.25 (1.25) | 4.28 (1.15) | 0.98 | 0.67, 1.43 | 0.91 | 0.51 |
| 20:3n-6/18:2n-6 | 0.05 (0.01) | 0.05 (0.01) | 3113 | 0, 3.8x10^18^ | 0.65 | 0.53 |
| 18:1n-9/18:0 | 2.85 (0.43) | 3.00 (0.52) | 0.53 | 0.20, 1.40 | 0.20 | 0.57 |
| 18:0/16:0 | 0.30 (0.03) | 0.29 (0.03) | 16.4 | 0, 1.2 x10^7^ | 0.68 | 0.53 |

**Supplemental Figure 1** Overall study design. FET= frozen embryo transfer, hCG = human chorionic gonadotrophin, LH= luteinising hormone, LMP= last menstrual period. ↑ = blood sampling. The current analysis presented here uses baseline (mean of 3.4 days prior; range 12 days prior to 1 day post) and day of FET (mean [standard deviation] 2.8 [0.9] days post-LH surge) measures to predict pregnancy success at day 47 post-LH surge.

**Supplemental Figure 2** Consort diagram for women recruited to the prospective study of ongoing pregnancy after natural cycle frozen embryo transfer *in vitro* fertilisation treatment.

196

consented

FET natural menstrual cycle

consented

35

withdrawn

3 withdrew consent

7 lost to follow up

25 cycles cancelled or constructed

161

IVF cycles

18

(2 successful on second or later attempt in the study; 16 failed)

repeat attempt cycles

143

unique first attempt IVF cycles

36

successful pregnancy

107

failed pregnancy

**Supplemental Figure 3. Relationship between saturated and unsaturated fatty acid concentrations.** Plots of erythrocyte (A and B) and plasma (C and D) unsaturated versus saturated fatty acid concentration at baseline (A and C) and at day of FET (B and D).

**Supplemental Figure 4** Baseline erythrocyte saturated to unsaturated fatty acid ratio in non-pregnant and pregnant groups. Median and interquartile range are shown. Difference testing by two sample t-test indicates the means are significantly different, P<0.001
